# Evaluation of a Laparoscopic Multi-approach Training for Obstetrics and Gynecology Residents

**DOI:** 10.1055/s-0040-1712997

**Published:** 2020-06-19

**Authors:** Débora Davalos Albuquerque Maranhao, Gustavo Anderman Silva Barison, Vanessa Alvarenga-Bezerra, Laís Assenheimer de Paula Ferreira, Anucha Andrade Schindler Leal, Renato Moretti-Marques, Sergio Podgaec, Eduardo Zlotnik, Mariano Tamura Vieira Gomes

**Affiliations:** 1 Department of Fetal-Matern Medicine, Hospital Israelita Albert Einstein, São Paulo, SP, Brazil

**Keywords:** medical education, laparoscopic surgery, residency, training, educação médica, cirurgia laparoscópica, residência médica, treinamento

## Abstract

**Objective** To analyze the applicability and efficiency of a multi-approach laparoscopic training in improving basic laparoscopic skills of obstetrics and gynecology (OBGYN) residents.

**Methods** Cross-sectional, observational and descriptive study, developed at the Experimentation and Surgery Training Center (CETEC, in the Portuguese acronym) of the Hospital Israelita Albert Einstein with OBGYN residents. Theoretical and practical tests were applied to 24 OBGYN residents to assess their laparoscopic skills before and after their participation in an 8-week course. The course involved theoretical lectures and practical laparoscopic surgery exercises developed using rubber models, black boxes, virtual simulators and animal models (pigs).

**Results** There was an overall improvement in the ability of the residents, with an increase in the number of correct answers in the theoretical evaluation and decrease in the time needed to perform practical tests (needle holder assembly and laparoscopic node). The course was evaluated by the students as highly relevant for both improving their surgical skills and motivating them to continue practicing.

**Conclusion** Laparoscopic training using multiple approaches resulted in significant improvement of surgical skills with a high satisfaction level of the participants. Further studies are still needed to measure the long-term retention of these acquired skills.

## Resumo

**Objetivo** Analisar a aplicabilidade e eficiência de um treinamento em laparoscopia com múltiplas abordagens, em melhorar as habilidades laparoscópicas básicas de residentes de ginecologia e obstetrícia (GO).

**Métodos** Estudo transversal, observacional e descritivo, desenvolvido no Centro de Treinamento em Experimentação e Cirurgia (CETEC) do Instituto de Pesquisa do Hospital Albert Einstein com residentes de GO. Foram aplicadas avaliações teóricas e práticas a 24 residentes de GO com o objetivo de avaliar suas habilidades laparoscópicas antes e após sua participação em um curso de 8 semanas. O curso envolveu palestras teóricas e exercícios práticos de cirurgia laparoscópica através de modelos de borracha, caixas pretas, simuladores virtuais e modelos animais (porcos).

**Resultados** Houve uma melhora geral na habilidade dos residentes, com aumento do número de respostas corretas na avaliação teórica e diminuição do tempo na execução dos testes práticos (montagem do porta-agulha e realização de nó laparoscópico). O curso foi avaliado pelos alunos como altamente relevante por melhorar suas habilidades cirúrgicas e motivá-los a continuar praticando.

**Conclusão** O treinamento laparoscópico utilizando múltiplas abordagens resultou em melhora significativa das habilidades cirúrgicas atrelado a alto nível de satisfação dos participantes. Novos estudos ainda são necessários para mensurar a retenção destas habilidades adquiridas a longo prazo.

## Introduction

Throughout past decades, laparoscopy (LP) became the first access choice for different procedures and specialties, as it provides, among other advantages, faster recovery, less pain and lower hospitalization length to the patients, in a way that there has been an increase in the use of minimally invasive access routes in surgery.^1^ ^2^

To be able to perform a laparoscopic surgery, a learning curve is required, as there is a need to develop and improve nonintuitive psychomotor functions, such as performing tasks under indirect vision and proprioception and handling with laparoscopic graspers.^3^ ^4^ It is crucial to all learning surgeons to practice these basic abilities before applying them to their patients. Therefore, laparoscopic training courses have gained great importance in the academic set.^1^

Different models are used during practice and some of them are low-cost models, such as rubber models and indirect vision boxes; others have higher cost, such as animals, virtual simulators and human cadavers. It seems to be consensus in the literature that the most technological or complex ones are as effective as simpler models, when the main objective is the development of the majority of the tasks, except for transferring objects between graspers or reproducing realistic scenarios.^5^ ^6^ ^7^

As gynecology is considered a surgical specialty, residents need to learn LP skills to keep up with current standards, so their training is extremely important for their maturation as laparoscopic surgeons. The present study has the objective to expose a laparoscopic multi-approach training course and to evaluate the improvement in laparoscopic skills in obstetrics and gynecology (OBGYN) residents, after attending it.^8^

## Methods

The present study is a transversal, observational and descriptive study, in which 24 residents were enrolled; 12 from the Hospital Israelita Albert Einstein (HIAE, in the Portuguese acronym) and 12 from the Hospital do Servidor Público Estadual de São Paulo (IAMSPE, in the Portuguese acronym). Students were selected according to the following criteria:

- Inclusion criteria: second and third year medical OBGYN residency students from the HIAE and the IAMSPE, who completed the video-laparoscopic course from November 2017 to November 2018; with minimum attendance of 80%.
- Exclusion criteria: first year OBGYN students from the HIAE and the IAMSPE; students with absence of 20% or more during the 2-month course period; who were absent on test application days; direct or indirect participants in the current study.

Between November 2017 and November 2018, all participants attended the LP course, following the previously outlined medical residency schedule of the two institutions, with 4 different participants every 2 months (i.e., 4 participants in November-December 2017; 4 participants in January-February 2018; and so on until September-October 2018). The entire course was applied by the same instructors, all of them with previous training in LP and technical proficiency in all skills taught.

Activities were developed at the Experimentation and Surgery Training Center (CETEC, in the Portuguese acronym) of the Instituto Israelita Albert Einstein, following these specifications: the same training was performed on 2^nd^ and 3^rd^ grade residents, with no progression of tasks; duration of 40 hours, divided into weekly activities for 2 months: 4 theoretical lectures about equipment, instruments and laparoscopic techniques, with a total length of eight hours; 16 hours of box-training; 8 hours of virtual laparoscopic simulator training; and 16 hours of animal model surgery training performed on pigs.

At the end of the training, it was expected that the student should be able to name the equipment needed for laparoscopic surgery and understand its basic operation and parameter setting. Besides that, the student should have learned how to properly handle the camera and optic, acquire spatial and proprioception notion while using graspers and know how to perform the first punctures, as well as the abdominal inventory. All these skills are compatible with the Brazilian Federation of Gynecology and Obstetrics (FEBRASGO, in the Portuguese acronym)^9^ competency matrix to a 2^nd^ year resident. The 3^rd^ year residents should even be able to perform simple animal model procedures.

During box training, students practiced camera proprioception exercises and visual-spatial orientation with bean grains, elastics and matches. Participants were always encouraged to use both hands and basic laparoscopic suture exercises were intensely practiced (adjusting the needle in the laparoscopic needle holder inside the video box, cross stitch and simple knot).

At the virtual simulator training, students had a sequence of programmed exercises, which included proprioception techniques, cavity inventory using the camera, bimanual skills, and surgical techniques for basic gynecological procedures such as tubal ligation. Unfortunately, as there was only one simulator, the training time on it was reduced in comparison to the other training modalities and, therefore, no specific evaluation using it has been included in the present study.

Finally, in animal model practicing, groups of 4 participants performed laparoscopic surgery on pigs. In this training, the tasks were: setting of the equipment and initial puncture, pneumoperitoneum and access to the peritoneal cavity, cavity inventory, surgical procedures such as lifting of the bladder, hysterectomy, bladder incision and suture. To make the most of the animal model, lymphadenectomy and rectosigmoidectomy were also performed, both focusing on the training of basic concepts and not the surgery itself, with the main objective of gaining greater sensitivity with the mobilization of living tissues using graspers.

**Fig. 1:**
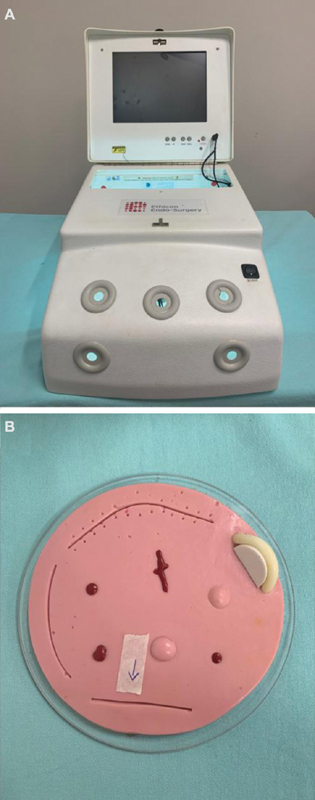
(**A**) “Black box” used for indirect vision training. (**B**) Rubber model for suture training.

The box used had the following specifications: 38.5 cm depth × 29.5 cm width (base), 32 cm (depth) × 29.5 cm width (top) × 20 cm height (Fig. 1A). Other equipment used consisted of 30° and 10 mm cameras, light source, LCD monitor, rubber models (Fig. 1B) to practice suture and knots and the laparoscopic simulator used was the LapVR laparoscopic surgical simulator (CAE Healthcare, Sarasota, FL, USA) .

All participants were evaluated individually using the same theoretical test (verbal questionnaire) and practical test, which were applied on the first and last day of the course (pre- and post-test, respectively). The students were not differentiated or compared between them, the only comparison made was the student with himself in the pre- and post-test.

The theoretical test consisted of two questions that included general knowledge about instruments and surgical equipment used in laparoscopic surgeries. The first question was: “What equipment and instruments need to be present in the operating room for LP?”, and it comprised a checklist of 10 items. Participants verbally listed the items, and for each correct answer in the checklist, the examiner granted them 1 point (maximum score of 10 points).

**Fig. 2:**
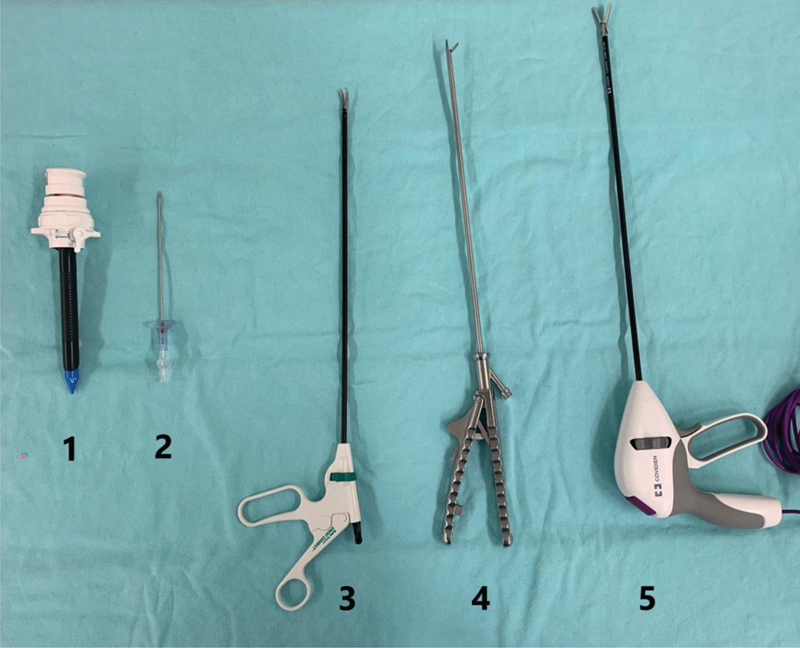
Equipment to be nominated. **1.** 10 mm trocater. **2.** Veress needle. **3.** Maryland or Dissecting laparoscopic forceps. **4.** Laparoscopic needle holder. **5.** Advanced bipolar.

The second theoretical question was to name five laparoscopic instruments placed on a table (Fig. 2). For each correct answer, the participant received one point, with a maximum of five points.

The practical test evaluated assembly of the needle in the needle holder and surgical suture, execution of a complete knot (three semi-knots) with needle support in a rubber model and transfer of three small plaster cylinders from one nail to another (removal of the cylinder from a nail with the laparoscopic grasper of the left hand, passing it to the right hand grasper in the air and placing it on another nail).

Most practical training evaluation studies in the literature use time-based performance to complete specific tasks known to analyze the skill level of the operator.^2^ ^5^ Measuring and comparing the performance time proved to be more effective and less subjective than using the judgement of an experienced evaluator on the quality of the task performed;^10^ therefore, we decided to evaluate the practical tests by time performance.

To ensure the uniformity of the evaluation, the researchers followed a pre-established checklist and were previously prepared on how to apply those tests, in a way that they were homogeneously applied. The scores of the questionnaires and practical tests were based on the number of correct answers and the time of completion of the proposed tasks.

A maximum time for conclusion of each question was stablished to avoid the test from becoming too extensive, and it was defined as ∼ 3 times the time spent by the course professors to do the same tasks (120 seconds – for the first practical task, 180 seconds for the second, and 300 seconds for the third). If the resident could not complete the task within this time, it was used the maximum seconds possible to calculate the results.

In addition, a global evaluation questionnaire of the course was applied on its final day (8 multiple choice questions) to provide a qualitative analysis of the course from the student perspective. The eight questions were:

1. Have you ever practiced LP in college or at any course in your life?
2. If yes: Have you practiced in simulators? Have you practiced in black boxes?
3. At the beginning of the course how would you rate your LP skills? Very poor, regular, good or very good.
4. Now, at the end of the course how would you rate your LP skills? Very poor, regular, good or very good.
5. How would you rate the course relevance to your LP learning ability? Irrelevant, slightly relevant or very relevant.
6. How effective was the course for you learning? Not effective, slightly effective, very effective.
7. How motivated have you been to practice LP after the course? Not motivated, slightly motivated or very motivated.
8. How satisfied are you with the course? Not satisfied, slightly satisfied or very satisfied.

Results and scores obtained from the questionnaires and practical tests were compiled and organized in specific tables, correlated by means of statistical analysis, with a significance level (*p*) of 0,05. Comparisons between pre- and post-test questions were made through the application of the Wilcoxon test.

The project was approved by the Ethics Committee for Research with Human Beings of the institution (submission number CAAE: 75234817.2.0000.0071). The participants were informed about the importance and purpose of the research and all those who agreed to participate signed a consent form.

The animal procedures were performed in accordance with the Brazilian Society of Science in Laboratory Animals norms, following the care recommended by the Assessment Association and Laboratory Accreditation of Animal Care and the Normative Instruction No. 7 of the Biosafety Commission. The release of animal use for this project by the Animal Research Ethic Committee was linked to the prior release granted to the scheduled LP course of the medical residency since 2015 and renewed annually by the Instituição Israelita Albert Einstein.

## Results

**Fig. 3:**
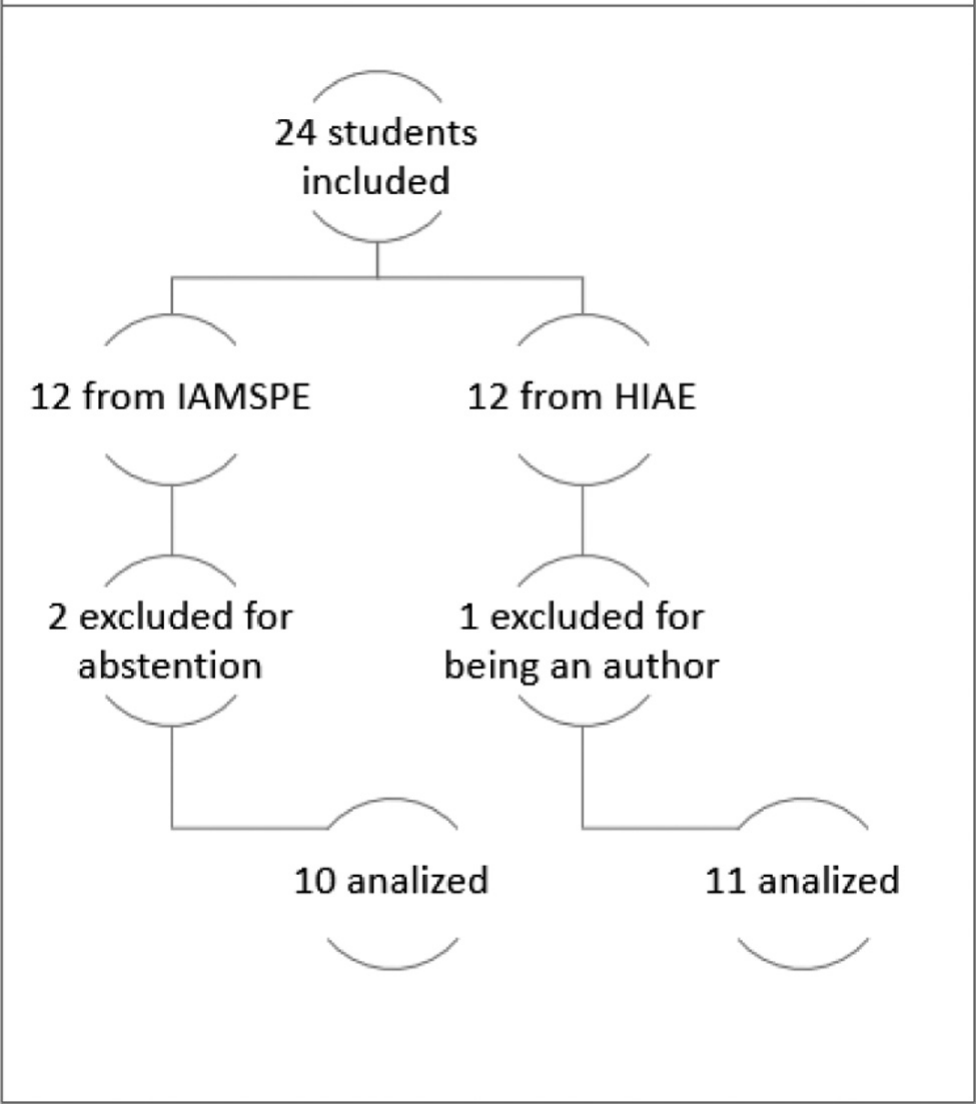
Participant selection workflow.

After applying the criteria, 24 students were included in the study and answered the tests, but 3 of them were posteriorly excluded, 1 for being one of the authors of the study, and 2 students due to absence in the post-course test; therefore, the final analysis was conducted with a total of 21 participants, as shown in the workflow of selection (Fig. 3).

The participants were all female, with an average age of 27 years old, physicians and OBGYN residents from the HIAE or the IAMSPE. The results showed improvement in post-test compared with pretest from most students in almost all questions. The questions made to students in the pre- and post-test are presented below:

- Question 1 (Theoretical) - Which equipments and instruments need to be present in the operating room for laparoscopy?
- Question 2 (Theoretical) - Nominate the instruments placed on the table.
- Question 3 (Practical) - Needle holder assembly and suture passage in rubber model.
- Question 4 (Practical) - Performance of a laparoscopic knot (three semi knots).
- Question 5 (Practical) - Transferring the cylindrical object from one nail to the other, passing directly between the laparoscopic graspers for three consecutive times.

The mean score of the pre-test for the first theoretical question was 5.81, with a median of 7.0, while the mean post test score was 8.45 with a median of 8.0. The difference was statistically significant in the post test with *p <* 0.001.

The same occurred with the second theoretical question. The mean score of the pre-test was 3.81 with a median of 4.0, compared with a mean post-test score of 4.76 and a median of 5.00, a difference also statistically significant, with *p =* 0.001.

It was observed a reduction in the time to complete all practical questions whose evaluations were time-based. The results were:

1) In question 3, we observed a statistically significant decrease of ∼ 44 seconds in the needle holder assembly and suture passage time in the post-test (mean of 36.14 and median of 32 seconds) in relation to the pretest (mean of 80.05 and median of 74 seconds), with *p <* 0.001.
2) Question 4 showed a decrease of 46 seconds in the average time for performing a complete laparoscopic node, with statistical significance (*p <* 0.001), comparing the pre-test (mean of 158.33 and median of 180 seconds) with the post-test (mean of 112.71 and median of 109 seconds).
3) In question 5, there was a reduction of 63 seconds in the mean time required to perform the task of transferring the cylindrical object. The pretest mean time was 213.81 seconds and median of 255 seconds, compared with the post-test (mean time of 150.29 seconds and median of 145 seconds), *p =* 0.048.

The mean, median and standard deviation (SD) that were calculated for each question and the comparative results after the Wilcoxon paired test are shown in Table 1. The nonparametric Wilcoxon test is indicated to compare two groups of information with numerical measurement level and paired samples, when we do not want to make assumptions about the distribution of the analyzed samples. It is specially indicated for studies with small samples.

**Table 1:** Statistical analysis with Wilcoxon Test

		Pre	Post	Wilcoxon Test (*p*)	Results
	Mean	5.81	8.48		
**Question 1**	Median	7	8	< 0.001*	Pre < Post
	Standard Deviation	2.34	0.87		
	n	21	21		
	Mean	3.81	4.76		
**Question 2**	Median	4	5	0.001*	Pre < Post
	Standard Deviation	1.08	0.44		
	n	21	21		
	Mean	80.05	36.14		
**Question 3**	Median	74	32	< 0.001**	Pre > Post
	Standard Deviation	39.8	21.77		
	n	21	21		
	Mean	158.33	112.71		
**Question 4**	Median	180	109	< 0.001**	Pre > Post
	Standard Deviation	36.88	52.16		
	n	21	21		
	Mean	213.81	150.29		
**Question 5**	Median	255	145	0.048**	Pre > Post
	Standard Deviation	90.42	83.83		
	n	21	21		

* Questions with higher significantly post-test answers.

** Questions 3, 4 and 5 had significantly lower post-test responses.

**Fig. 4:**
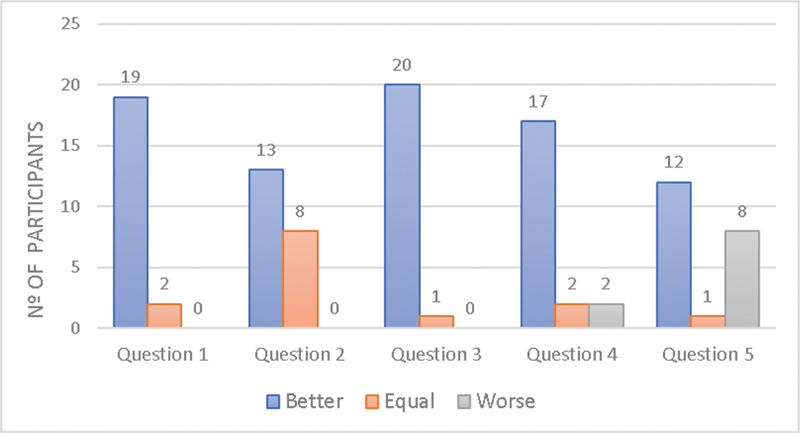
Number of participants that were Better, Equal or Worse in each question comparing post with pre tests.

In all questions, most of the participants got better results when comparing the post-test with the pretest, except for questions 4 and 5, in which some students had worse results at the post-test, as shown in Fig. 4.

Because the qualitative evaluation of the course was optional and was requested on the last day of training, only 10 participants answered the questionnaire. Overall, the answers given reflected a good acceptance of the course, with all students reporting surgical improvement on their laparoscopic ability at the end of the training. Most students classified the course as very relevant, and all of them answered that they were more motivated to practice and improve their skills after it.

## Discussion

In the present study, we evaluated the effectiveness of a laparoscopic training course that complied with some pillars designed initially by Sun et al.^11^ The authors established the following pillars for a favorable learning curve to laparoscopic training: (a) laboratory skills training; (b) training in pelvic models or black boxes; (c) animal models; (d) supervised clinical training; (e) selection of cases to be treated first.

Following these principles, participants attended theoretical lectures, practical activities in rubber models, training in black boxes and animal surgery, always supervised by tutors and subsequently submitted to learning evaluation.^11^

The main results demonstrate a significant improvement in both theoretical and practical skills of all students after a 2-month laparoscopic course, which is consistent with similar results found in the literature for similar trainings.^12^

In the study by Derossis et al,^13^ 4 out of 7 evaluated laparoscopic practice tasks had significantly better results after developing a similar training with doctors. Andreatta et al^14^ also presented better results when comparing interns who underwent virtual simulator training with a control group not trained at all. The evaluation was applied after a practice surgery on pigs, and the group that trained on the simulator achieved greater accuracy on camera navigation and in the transfer of objects between graspers.^14^

Some literature studies compared educational techniques in LP, such as the one made in the Hong Kong Academy of Medicine, that applied a course with different training techniques, supposedly complementary, with the purpose to diversify and create a more effective learning by stimulating different skills at the same time.^8^ ^15^

The qualitative analysis of Ko et al,^8^ conducted after the overall assessment of the students, showed a high satisfaction rate with the course and a sense of technical improvement. However, there were no significant differences between students who practiced in simulators with those who practiced in black boxes.^8^

A Brazilian study conducted by the Universidade Federal de São Paulo/Escola Paulista de Medicina (UNIFESP/EPM, in the Portuguese acronym), also demonstrated significant improvement in the competence in students sense of competence to perform level 1 surgeries (diagnostic LP and tubal ligation) and level 2 surgeries (ovarian biopsy, lysis of adhesions, oophorectomy and ectopic pregnancy) after attending a practical course, with an unanimous training approval.^16^

A strength of our study is that this training is inserted as a counterpoint to Brazilian reality, as the country experiences contrasts in medical education, so that in one hand, few large universities are able to invest sources in training to qualify their students,^15^ while in the other hand, others deal with lack of adequate institutional structure and low quality technology, due to low funds.^1^

Following this scenario, it is erroneously believed that only high-tech and high-cost courses would be suitable for learning. In our laparoscopic course, we managed to combine purposely high-tech methods with low-cost and poor-technological models, to reduce costs while maintaining quality. There are studies in the literature that explore inexpensive models for successful surgical training, as the one developed by Sharma et al,^4^ in which the students performed self-LP-training by building black boxes using computer cameras to develop basic skills, at a minimal cost.

Other new technologies are being studied to improve learning and reduce costs, such as what has been demonstrated by Vyas et al^17^ through the development of a laparoscopic simulator that uses cellphones and laptops to train surgeons, with very satisfactory results. According to Chalhoub et al,^18^ even cellphone applications can be used to improve laparoscopic skills.

In contrast, given the complexity of real-life surgeries, one of the limitations of our study is that simple model training is not always enough for the formation of the surgeon. An alternative to the animal model, known for being closer to reality, are cadavers, an important complementary step before in vivo practice for enabling and amplifying basic techniques.^19^ ^20^

Another point that was not accomplished in the present study is the access to the long-term knowledge retention of the students, as we apply the post-test as soon as they finish the training; also, it is not possible to affirm the effectiveness of the course for their application in vivo, because there was no evaluation on real surgeries.

Different training courses are being developed with the proposal of improving the long term learning retention of practical skills, as the “spaced learning training in LP,” where the time training is broken with 20 minutes of active distractions, and it showed improvement in time and quality of tasks, even after a long-term test, when comparing spaced to traditional training. This can be a good alternative for future studies.^21^

As limiting factors of the present study, we can highlight the absence of a control group, a restricted number of students and an exclusive time performance evaluation of practical skills, even though time is still an important parameter used in similar studies.^22^ ^23^ Repeating the same previously familiar task may also have increased the chance of correct answers in the post-test.

We know that training young surgeons is crucial to reduce errors and improve skills that will be later used in our patients, so that a multi-approach training course, like the one we presented, could maybe reduce costs without losing any quality on teaching and become a model for universities and medical services that still do not have this sort of training program on their residency schedule. Another important key is to have an assessment tool like the pre and post-test to measure the student improvement immediately after the course.

## Conclusion

The study showed that the multiple-teaching approach to LP training resulted in a significant technical improvement in students’ skills on the asked tasks. Theoretical knowledge and practical skills were both improved through training, in accordance with the literature, suggesting that this acquired learning can be expressed in the future with the improvement of surgical ability and confidence in the execution of surgical procedures in vivo.
